# The Role of Polymorphisms at the Interleukin-1, Interleukin-4, GATA-3 and Cyclooxygenase-2 Genes in Non-Surgical Periodontal Therapy

**DOI:** 10.3390/ijms23137266

**Published:** 2022-06-30

**Authors:** Kay-Arne Walther, José Roberto Gonzales, Sabine Gröger, Benjamin Ehmke, Dogan Kaner, Katrin Lorenz, Peter Eickholz, Thomas Kocher, Ti-Sun Kim, Ulrich Schlagenhauf, Raphael Koch, Jörg Meyle

**Affiliations:** 1 Department of Periodontology, University of Giessen, Schlangenzahl 14, 35392 Giessen, Germany; office@prof-gonzales.de (J.R.G.); sabine.e.groeger@dentist.med.uni-giessen.de (S.G.); joerg.meyle@dentist.med.uni-giessen.de (J.M.); 2 Department of Periodontology, University of Münster, Albert-Schweitzer-Campus 1, Waldeyerstraße30, 48149 Münster, Germany; ehmke@uni-muenster.de; 3 Departments of Periodontology and Synoptic Dentistry, Charite Centrum 3, Charite-Universitatsmedizin Berlin, Aßmannshauserstraße 4-6, 14197 Berlin, Germany; dogan.kaner@uni-wh.de; 4 Department of Periodontology, University of Witten/Herdecke, Alfred-Herrhausen-Straße 44, 58455 Witten, Germany; 5 Department of Periodontology, TU Dresden, Fetscherstraße 74, 01307 Dresden, Germany; katrin.lorenz@tu-dresden.de; 6 Department of Periodontology, Johann Wolfgang Goethe-University Frankfurt, Theodor-Stern-Kai 7, 60596 Frankfurt, Germany; eickholz@med.uni-frankfurt.de; 7 Unit of Periodontology, University of Greifswald, Rotgerberstraße 8, 17475 Greifswald, Germany; kocher@uni-greifswald.de; 8 Section of Periodontology, Department of Conservative Dentistry, University of Heidelberg, Im Neuenheimer Feld 400, 69120 Heidelberg, Germany; tisun.kim@med.uni-heidelberg.de; 9 Department of Periodontology, University of Würzburg, Pleicherwall 2, 97070 Würzburg, Germany; schlagenha_u@ukw.de; 10 Institute of Biostatistics and Clinical Research, University of Münster, Schmeddingstraße 56, 48149 Münster, Germany; raphael.koch@ukmuenster.de

**Keywords:** periodontitis, polymorphisms, risk factor, periodontal therapy, antibiotics, GATA-3, Interleukin-1, Interleukin-4, Cyclooxygenase-2

## Abstract

Periodontitis is a multifactorial disease. The aim of this explorative study was to investigate the role of Interleukin-(IL)-1, IL-4, GATA-3 and Cyclooxygenase-(COX)-2 polymorphisms after non-surgical periodontal therapy with adjunctive systemic antibiotics (amoxicillin/metronidazole) and subsequent maintenance in a Caucasian population. Analyses were performed using blood samples from periodontitis patients of a multi-center trial (ClinicalTrials.gov NCT00707369=ABPARO-study). Polymorphisms were analyzed using quantitative real-time PCR. Clinical attachment levels (CAL), percentage of sites showing further attachment loss (PSAL) ≥1.3 mm, bleeding on probing (BOP) and plaque score were assessed. Exploratory statistical analysis was performed. A total of 209 samples were genotyped. Patients carrying heterozygous genotypes and single-nucleotide-polymorphisms (SNP) on the GATA-3-IVS4 +1468 gene locus showed less CAL loss than patients carrying wild type. Heterozygous genotypes and SNPs on the IL-1A-889, IL-1B +3954, IL-4-34, IL-4-590, GATA-3-IVS4 +1468 and COX-2-1195 gene loci did not influence CAL. In multivariate analysis, CAL was lower in patients carrying GATA-3 heterozygous genotypes and SNPs than those carrying wild-types. For the first time, effects of different genotypes were analyzed in periodontitis progression after periodontal therapy and during supportive treatment using systemic antibiotics demonstrating a slight association of GATA-3 gene locus with CAL. This result suggests that GATA-3 genotypes are a contributory but non-essential risk factor for periodontal disease progression.

## 1. Introduction

Periodontitis is a multifactorial disease accompanied by attachment loss which finally results in tooth loss. It is initiated by a dysbiotic biofilm which elicits a destructive inflammatory response. Periodontitis is clinically classified into staging, grading, extent and distribution. The latest classification of periodontal diseases addresses to future research the identification of specific genetic markers to differentiate between distinct periodontitis phenotypes. This could reflect clinically the initiation and progression of periodontitis [1,2]. It is known that genetic factors are involved in the pathogenesis of periodontitis [3,4,5,6,7,8]. However, there are limited data on the role of genetic factors in disease progression [9,10,11]. Genetic factors and environmental modifiers are known to influence disease severity. Analysis of disease expression in twins showed that a considerable variance in clinical phenotype is explained by genetic factors [3,4]. The IL-1 cytokine family has key regulatory roles in innate and adaptive immunity. Variations in IL-1 genes were first associated with chronic periodontitis (CP) in Caucasians in 1997 [12]. Many studies have subsequently explored the role of IL-1 gene polymorphisms in periodontitis with mixed results [13,14,15,16,17]. A couple of studies evaluated the influence of IL-1 SNP on the progression of periodontitis with a small sample size, partially with a short observation period and different results [9,10,18,19,20,21,22].

IL-4 is a multifunctional cytokine which induces polyclonal B-cell proliferation. IL-4 differentiates naive CD4+ T cells to TH2 cells, which in turn produce IL-4 [23,24]. A meta-analysis by Yan et al. [25] showed a significant association between CP patients and the T/T genotype at position IL-4 −590 in Caucasians, whereas a recent meta-analysis by a Bayesian approach found no association between CP and IL-4 −590 [17]. The gene loci IL-4 −590 and IL-4 −34 are in linkage disequilibrium which has been demonstrated in CP [26] and in aggressive periodontitis (AgP) patients [27].

GATA factors are pleiotropic transcription factors of the C4 Zinc finger family. GATA-3 is present in early thymocyte cells. Thereby, it is a key transcription factor for gene expression in the single stages of TH2 cell differentiation [28]. GATA-3 expression is induced by IL-4 in a STAT-6-dependent signaling pathway. For STAT-6 deficiency, GATA-3 can continue the TH2 differentiation. If GATA-3 is absent, T cell differentiation into TH2 cells is disturbed [29].

To date, there are only few GATA-3 SNPs reported. A well-documented locus is on the gene locus rs3802604. This locus was associated with type 1 diabetes mellitus [30], a significant reduction in breast cancer risk [31] and a higher relapse-free survival rate [32].

COX-2 is produced mainly in inflammatory processes [33]. Many studies have highlighted the importance of arachidone derivatives on the progression of periodontal disease. In particular, prostaglandin E2 and leukotriene B4 are involved in periodontal destruction. They are strongly increased in the inflamed periodontal tissue and in the crevicular fluid in gingivitis, periodontitis and peri-implantitis patients [34,35,36,37,38]. Interesting results were published regarding the gene locus COX-2 −1195 (rs689466). SNP on this position was associated with CP in a Chinese population [39]. On the contrary, there was no association in the European population [40].

The present study investigated genetic factors from patient samples with periodontitis that participated in the placebo-controlled, multi-center ABPARO trial (ClinicalTrials.gov NCT00707369). Clinical, microbiological and systemic results have been previously reported [41,42,43,44,45].

The aim of the present exploratory subanalysis was to evaluate the role of the IL-1A −889, IL-1B +3954, IL-4 −34, IL-4 −590, GATA-3 IVS4 +1468 and COX-2 −1195 gene loci in patients with periodontitis after a non-surgical periodontal therapy with or without additional adjunctive systemic antibiotics.

## 2. Results

In the present study the results of the genetic analyses of the gene loci at positions IL-1A −889 (rs1800587), IL-1B +3954 (rs1143634), IL-4 −34 (rs2070874), IL-4 −590 (rs2243250), GATA-3 IVS4 +1468 (rs3802604) and COX-2 −1195 (rs689466) are shown. The results of the clinical and microbiological parameters have been demonstrated in previous publications [41,42,43,44,45].

A total number of 209 FTA elute micro cards was genotyped. The patient characteristics and the smoking status of all individuals are shown in Table 1. The external and internal validation of the genetic analyses did not show abnormalities. Genotype frequencies of the analyzed collectives are presented in Table 2. All genotypes were presented in a sufficient number of individuals to perform the analyses.

Table 3 shows the changes of the clinical attachment level (CAL) from month 27.5 (visit 12)-baseline (visit 2) and from month 27.5 (visit 12)-month 3.5 (visit 4) in the total group of individuals, the placebo group and in the antibiotics group, with their respective *p*-values.

With regard to the GATA-3 genotypes, the results presented in Table 3 are summarized as follow: after 27.5 months, changes in CAL (between visit 12 and visit 2) and changes between visit 12 and visit 4 were larger in individuals presenting the GATA-3 SNP (A/A) than GATA-3 wild-type (G/G), which means that between baseline and 27.5 months there was a greater CAL gain in GATA-3 SNP (A/A) individuals than in wild-type individuals, while wild-type patients showed more CAL loss after the 3.5 months visit than GATA-3 SNP (A/A) patients. In the total group and in the placebo group, there was a higher attachment loss for the wild-type patients (*p* < 0.05). In addition, in the total group, the GATA-3 heterozygous patients showed less CAL loss after 27.5 months (between visit 12 and visit 4) than wild-type patients (*p* = 0.0473). With regard to the IL-1A genotypes, there was higher gain of CAL after 6 months (between visit 6 and visit 2) in the total group of IL-1A −889 wild-type patients compared to heterozygous patients (*p* = 0.0170). In the placebo group, there was a higher CAL gain after 27.5 months (between visit 12 and visit 4) in the heterozygous patients versus the wild-type patients (*p* = 0.0444). In contrast, CAL loss was higher after 27.5 months (between visit 12 and visit 2 and between visit 12 and visit 4, respectively) in the groups of patients carrying the IL-1B +3954 wild type compared to patients with heterozygous and SNP genotypes, but not statistically noticeably different (*p* > 0.05).

In addition, there was a higher CAL loss after 27.5 months (between visit 12 and visit 4) between the patients carrying the SNP versus the wild-type patients in the antibiotics group (*p* = 0.0427). In the placebo group, differences between the genotypes were smaller.

After 27.5 months, changes in BOP (Table 4) and plaque scores (Table 5) (between visit 12 and visit 2 as well as between visit 12 and visit 4) were not influenced by genotypes of the analyzed gene loci.

The multivariable linear regression model confirmed the univariate analysis to explain CAL gain or loss after 27.5 months (Table 6). Individuals presenting with GATA-3 SNPs and heterozygous patients had statistically noticeably lower CAL loss over 27.5 months than wild-type patients. Patients carrying the GATA-3 SNP had −0.32 mm (95% CI: −0.57 mm, −0.07 mm) and heterozygous patients had −0.18 mm (95% CI: −0.43 mm, 0.07 mm) less change in the mean CAL than wild-type patients. In comparison, systemic antibiotic therapy leads to 0.22 mm (95% CI: 0.04 mm, 0.39 mm) more CAL improvement between 27.5 months and baseline than placebo.

**Figure 1:**
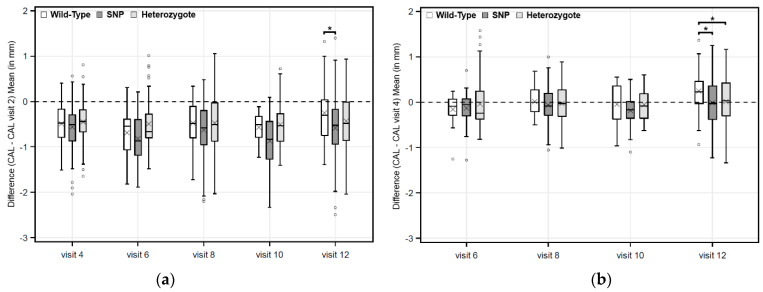
Boxplots of GATA-3: Mean CAL differences to visit 2 (**a**) and to visit 4 (**b**) in the total collective: * *p*-values are from Mann–Whitney U-tests for the pairwise comparison with the wild-type group. Negative values represent an improvement and positive values represent a deterioration. Abbreviations: CAL: mean clinical attachment level per patient, SNP: single nucleotide polymorphism, mm: millimeter, × marks the mean.

The IL-1A- 889 gene locus seems to influence CAL change (between visit 12 and visit 4) after 27.5 months. In addition, the figures of CAL change (between visit 12 and visit 2 respectively between visit 12 and visit 4) from the GATA-3 IVS4 +1468 gene loci are shown in Figure 1.

The percentage of sites per patient showing further attachment loss (PSAL) ≥1.3 mm showed similar results (results are shown in the supplementary file).

## 3. Discussion

In the present study, the influence of IL-1, IL-4, GATA-3 and COX-2 polymorphisms on the outcomes of non-surgical therapy with and without antibiotics and 2 years of maintenance was analyzed in Caucasian periodontitis patients. This is an exploratory subanalysis of a subset of the per-protocol collective from the prospective, randomized, double-blind, multi-center ABPARO trial (ClinicalTrials.gov NCT00707369) on the effect of adjunctive systemic amoxicillin 500 mg plus metronidazole 400 mg (3×/day, 7 days) [44,46]. The clinical results reported improvement in all clinical parameters after non-surgical periodontal therapy. Overall, after 27.5 months, the additional use of adjunctive antibiotics led to significantly better clinical results than mechanical debridement alone [44]. In severely diseased and younger patients, adjunctive antibiotics led to clinically relevant better improvements than placebo [42]. Nevertheless, the use of antibiotics should be carefully considered. Recent studies indicate that probiotics, paraprobiotics and postbiotics have a positive clinical effect on periodontitis remission [47,48]. This could be an alternative to reduce antibiotic medication.

In the last decades, some genetic markers which may be associated with the progression of the disease have been identified [12]. It is known that periodontitis is a polygenetic disease [49,50,51]. Individual genetic factors may modify the host immune response to the dysbiotic biofilm [7,52,53]. Associations between genetic factors, systemic diseases and lifestyle factors were also reported [54]. Meta-analyses of case-control studies demonstrated that SNPs were associated with periodontitis [14,15,16,17,25,55,56]. Only few studies demonstrated large effects of SNPs on the stage of disease [39,57,58].

This is the first study evaluating longitudinally the effect of 6 gene loci on changes in CAL, PSAL ≥1.3 mm, BOP and plaque scores following non-surgical periodontal treatment and 2 years of maintenance. The primary outcome variable of this subanalysis was the change of the mean CAL per patient between 27.5 months (visit 12) and baseline (visit 2), and between 27.5 months and 3.5 months (visit 4), respectively. Periodontitis progression was quantified by the paired comparison of the mean CAL of the 3 genotypes in 6 gene loci. In addition, the adjusted influence of the gene loci on disease progression was assessed by a multivariate analysis. A total of 209 patients (104 placebos, 105 antibiotics) were genotyped. Out of these patients, 136 could not be genotyped because the amount of sampled blood was insufficient.

The main results demonstrate differences for the CAL change between the genotypes. Patients carrying the GATA-3 SNP or the heterozygous genotype showed lower CAL loss during supportive periodontal therapy (SPT) in comparison with the patients carrying the wild-type. These data provide new insights since only few studies have investigated this genetic marker [30,31,32] and actually no periodontal-genetic clinical study was performed yet. Zhao et al. [59] analyzed the expression of Th17/Th1/Th2 cytokines and transcription factors, and Th17 cell vibration in Chinese CP patients. The expression of transcription factors (RORC, T-bet and GATA-3) in peripheral blood was measured by real-time PCR, and the levels of Th17 cells in CD4(+) T cells were analyzed by flow cytometry. In the mouse model, it was demonstrated that the expression of the Th2 differentiation maintaining transcription factor GATA-3 [28] in Th2 cells after treatment compared to the expression before treatment was increased 1.76-fold (*p* < 0.05). Intracellular staining of IL-17 revealed that the quantity of Th17 cells in contrast decreased (*p* < 0.05), especially the IL-17(+) IFN-γ(+) subgroup. The results indicated a protective effect of Th2 cells.

The results of the present study with regard to the IL-1 genotype are similar to the results of the study by Ehmke et al. [9] investigating the prognostic value of the IL-1 haplotype on the progression of periodontal disease following therapy. Forty-eight adult patients with untreated periodontitis harboring *Aggregatibacter actinomycetemcomitans* and/or *Porphyromonas gingivalis* were randomly assigned to receive full-mouth scaling alone (control) or in combination with systemic antibiotic treatment (metronidazole plus amoxicillin) and supragingival irrigation with chlorhexidine digluconate (test). All patients received SPT at 3 to 6-month intervals. In 33 patients, DNA was analyzed for polymorphism in the IL-1A gene at position −889 and IL-1B gene at position +3953. These results indicated that the IL-1 haplotype may be of limited value for the prognosis of periodontal disease progression following non-surgical therapy.

The study of Meisel et al. [21] evaluated the genetic influence of IL-1A, IL-1B and IL-1RN polymorphisms on periodontal variables in relation to environmental factors such as smoking. No differences were found in allele frequencies or combined allotypes between subjects with mild or moderate versus those with severe periodontitis. However, the extent of CAL loss defined as percentage of sites >4 mm was significantly associated with the composite genotype of IL-1A/-1B in smokers. The results provided evidence that the genotypes studied show interaction with smoking, the main exposition-related risk factor of periodontitis.

Eickholz et al. [10] analyzed patient-related factors contributing (1) to tooth loss and (2) to the quality of treatment outcomes 10 years after initiation of non-surgical therapy. All patients who had received active periodontal treatment 10 years ago by the same examiner were recruited until a total of 100 patients was re-examined. The following risk factors for tooth loss were identified: ineffective oral hygiene, irregular SPT, IL-1A/-1B polymorphism, initial diagnosis, smoking, age and sex.

In the present study, there were small or no differences in the change of CAL between the genotypes for the IL-1B +3954 gene locus. Wild-type patients showed higher improvement of CAL after non-surgical periodontal therapy than heterozygote or SNP patients. In the antibiotic group there was a difference in the change of the CAL between 27.5 months and 3.5 months between SNP versus wild-type patients (*p* = 0.0427).

Successful anti-infective therapy as well as SPT in short intervals [60] led to insignificant differences for BOP and plaque score between genotypes. With regard to the IL-1A −889 genotypes the median change in CAL (between 27.5 months and 3.5 months) observed in the placebo group was 0.0 mm (−0.5 mm, 0.2 mm) compared to 0.2 mm (−0.1 mm, 0.5 mm) between heterozygote versus wild-type patients (*p* = 0.0444). Therefore, it may be concluded that heterozygote patients had less CAL loss during 24 months of SPT. The multivariable linear regression models support these results.

Although many case-control studies have been conducted on the association of IL-4 with periodontitis [25], there is no longitudinal study that can be compared with the present analysis. Comparison of the clinical parameters in the IL-4 and COX-2 genotype showed no differences or indifferent results between the genotypes. The role of COX-2 in the progression of periodontitis has been previously reported. Important mediators are the end-products of the COX cycles such as prostaglandin, prostacycline, thromboxane and leukotrienes [61]. Mesa et al. [62] reported an association of higher COX-2 expression levels with CAL loss, BOP and loss of connective tissue in gingivitis and periodontitis patients. Beikler et al. [63] reported a significant reduction of the gene expression of COX-2 after non-surgical periodontal therapy.

Three case-control studies focused on the frequency of the COX-2 −1195 gene locus in periodontitis patients [39,40,64], including a Caucasian population [40]. The ethnologically comparable study of Schäfer et al. [40] with the present study did not identify an association between SNP at position −1195 and CP or AgP patients. In contrast, Xie et al. [39] demonstrated a significantly increased risk of CP in a Chinese population in the presence of SNP at position −1195. Daing et al. [64] confirmed these results in a North Indian population.

The secondary outcomes in the present study were PSAL ≥ 1.3 mm, BOP and plaque score. PSAL ≥ 1.3 mm demonstrated similar results as CAL. No difference in the change of BOP and plaque score was observed between the genotypes. Our results were in concordance with the data of Ehmke et al. (1999) [9] and Konig et al. (2005) [19] who analyzed both IL-1 gene loci from Caucasian CP patients. PSAL ≥ 2 mm and BOP showed no differences between the IL-1 genotypes 2 years after SPT [9]. Furthermore, no differences in pocket probing depths (PPD), tooth loss and plaque score could be demonstrated after 13 years of SPT [19].

The present study investigated the association of several known genetic factors in the pathogenesis of periodontitis with disease progression and the influence of systemic antibiotics. Young patients would benefit from an early diagnosis of severe periodontal disease. If many teeth had to be extracted in the early years of life due to periodontitis, additional complications will arise in the future, including rehabilitation by implants. Peri-implantitis has a more rapid disease progression than periodontitis [65] and today’s life expectancy for young people suggests multiple future implant placements at the same site. This creates a high level of physical and financial burden for the affected patients.

## 4. Materials and Methods

### 4.1. Study Design

The present study was designed as a subanalysis of the per-protocol collective data from the prospective, randomized, stratified, double-blind, multi-center trial (ClinicalTrials.gov NCT00707369) [46]. The results of the clinical study were previously reported [41,42,43,44,45]. The trial analyzed the effects of adjunctive systemic administration of amoxicillin 500 mg plus metronidazole 400 mg (3×/day, 7 days) on different periodontal parameters in patients suffering from moderate to severe periodontitis. Patients who followed the study timeline according to the protocol were included in the per-protocol collective.

Caucasian patients with untreated moderate to severe CP and AgP were included (periodontitis stage III or IV with grade B or C). Key inclusion criteria were: age (18–75 years), a Community Periodontal Index of Treatments Needs (CPITN) of IV in at least one sextant, at least 10 natural teeth in situ and PPD of ≥6 mm at a minimum of 4 teeth. Key exclusion criteria were: confirmed or assumed allergies or former hypersensitive skin reactions to amoxicillin and/or metronidazole, systemic medications affecting periodontal health and pregnancy. Type 2 diabetes mellitus was diagnosed by taking a blood sample and determining the HbA1c. Smoking status was checked by Bedfont-Smokerlyzer (Bedfont, UK) and the body mass index was calculated. Other systemic diseases were inquired by anamnesis (e.g., Down Syndrome, AIDS/HIV or systemic medication affecting periodontal conditions). For details, refer to Harks et al. [46].

Genetic evaluations were performed after closure of the ABPARO database [44], i.e., the laboratory staff were blinded to all dental measurements. All per-protocol patients in whom genotyping was possible were included.

The institutional review boards (IRB) of the participating centers approved the protocol and all patients had provided written informed consent. An independent data and safety monitoring board reviewed the safety data throughout the trial.

### 4.2. Periodontal Therapy

The study duration per patient comprised 12 visits over 27.5 months. Within 1.5 months after baseline examination (visit 2), patients received supra- and subgingival debridement in one or two sessions on one/two consecutive days (visit 3). All mechanical therapy was performed with hand instruments and/or machine-driven scalers. After completion of mechanical debridement, the antibiotic group of patients received two antibiotics (amoxicillin 3H_2_O 574 mg (Amoxicillin-ratiopharm 500 mg^®^, Ratiopharm, Ulm, Germany); metronidazole 400 mg (Flagyl^®^ 400, Sanofi-Aventis, Frankfurt, Germany)). The placebo group patients received two placebo tablets, each to be taken 3 times a day for 7 days. In addition, patients were instructed to rinse their mouth with Chlorhexidine 0.2% twice per day for 7 days. Furthermore, they were instructed to brush with their toothbrush 2 min twice per day at home applying a technique suitable for the individual. Additional oral hygiene devices, such as dental floss or interdental brushes, were recommended depending on the patient’s individual need.

Re-evaluation (visit 4) was performed 3.5 months after baseline and at least 2 months after mechanical debridement. Thereafter, all patients received SPT, including full-mouth supragingival debridement and oral hygiene instructions at 3-month intervals. Sites with PPD ≥ 4 mm also received subgingival re-debridement.

### 4.3. Examinations

Periodontal parameters were assessed at 6 sites of each tooth by blinded examiners not involved in the periodontal treatment. All measurements were performed at baseline (visit 2), 3.5 months (re-evaluation, visit 4) and at 27.5 months (visit 12). CAL was calculated by measurements of PPD and gingival recession that were performed with an electronic pressure-sensitive probe (Standard Florida Probe, Gainesville, FL, USA) in increments of 0.2 mm. The mean CAL (in mm) was calculated for each patient. The difference in the mean CAL between the 27.5 months (visit 12) to visit 2 and visit 4 described the changes of the CAL (gain or loss of tooth supporting tissue). After the X-ray appraisal and the evaluation of the anamnesis, the diagnosis was made according to the 1999s classification [66,67].

The primary outcome variable in this study was the change in mean CAL per patient and was assessed in an exploratory manner. Thus, the presence of attachment loss is representative for disease progression. In addition, the following secondary endpoints were included: PSAL ≥1.3 mm, percentage of sites per patient with BOP [68] and percentage of sites per patient showing supragingival plaque score [69]. BOP and Plaque scores were recorded at the baseline investigation (before subgingival instrumentation), at re-evaluation and during 24-month SPT (plaque score and BOP in a 3-month interval) (compare Harks et al. (2015) [44]). Differences in the percentage of BOP and in the percentage of supragingival plaque score per patient were calculated between 27.5 months (visit 12) and baseline (visit 2) or re-evaluation (visit 4).

### 4.4. Genotyping

Gene loci at positions: IL-1A −889 (rs1800587), IL-1B +3954 (rs1143634), IL-4 −34 (rs2070874), IL-4 −590 (rs2243250), GATA-3 IVS4 +1468 (rs3802604) and COX-2 −1195 (rs689466) were analyzed using qPCR. 345 FTA elute micro cards (GE Healthcare UK, Little Chalfont, UK) from patients were used to obtain genomic DNA. A Ø 1.25 mm Harris micro punch (GE Healthcare UK, Little Chalfont, UK) was used to excise 6 pieces from a FTA card. The pieces were placed into a micro centrifuge tube, added with 500 μL of dH2O and vortexed 3x for 5 s. Thereafter, the pipette was used to remove the water. After 5 s centrifugation, the DNA elution was performed in 50 μL dH2O at 95 °C for 30 min in a heating block. The quality of DNA was analyzed by NanoDrop 2000 (Thermo Fisher Scientific, Watham/MA, USA) UV-Vis Spectrophotometer and monitored for the values of OD260/OD280. Then, 1 μL of the DNA extract was used as template in each qPCR. The qPCR was performed with the TaqMan SNP Assay^®^, and the TaqMan Genotyping Master Mix^®^ (both Applied Biosystems Inc., Foster City/CA, USA) and analyzed using a Bio-Rad C1000 thermal cycler (Bio-Rad Laboratories Inc., Hercules/CA, USA). For validation of each SNP, one positive and two negative controls were included. Each sample was analyzed three times. The results of the TaqMan qPCR were verified by Sanger sequencing.

### 4.5. Statistical Analysis

Statistical analyses were performed using SAS software, version 9.4 of the SAS System for Windows (SAS Institute, Cary/NC, USA). Inferential statistics such as *p*-values and confidence intervals were intended to be exploratory, not confirmatory. The *p*-values represent a metric measure of evidence against the respective null hypothesis and were used only to generate new hypotheses. Therefore, neither global nor local significance levels were determined, and no adjustment for multiplicity was applied. Consequently, explorative two-sided *p*-values ≤ 0.05 were denoted as statistically noticeable instead of significant.

Sample size calculation was performed for the initial randomized ABPARO trial (compare [44]). In the exploratory analyses in this study, all per-protocol patients in whom genotyping was possible (*n* = 209) were included.

Standard univariate statistical analyses were performed to describe demographical and clinical characteristics. Categorical variables are shown as absolute and relative frequencies. Normally distributed continuous variables are shown as mean ± standard deviation. Not normally distributed continuous variables are reported as median (25% quantile–75% quantile). The relationship between the categorical variables was verified using Fisher’s exact test. Pairwise comparisons of outcome variables between wild type, heterozygous genotype and SNP were performed using two-sided nonparametric Mann–Whitney U-tests. In order to estimate the adjusted influence of the gene loci on the changes of the CAL, a multivariate analysis was performed by fitting a linear regression model. The dependent variables were the difference in the mean CAL per patient between visit 12 and 2 and the difference between visit 12 and 4. The influencing factors were the main effect of treatment group and of each gene loci: IL-1A −889 (rs1800587), IL-1B +3954 (rs1143634), IL-4 −34 (rs2070874), IL-4 −590 (rs2243250), GATA-3 IVS4 +1468 (rs3802604) and COX-2 -1195 (rs689466). Results are reported as regression coefficients and the corresponding 95% confidence interval (CI).

## 5. Conclusions

Within the limitations of this explorative analysis, the results of the present study demonstrate that specific genetic polymorphisms may have an impact on long-term progression of periodontitis. GATA-3 genotypes might be useful for predicting disease progression in severe periodontitis. Individuals with the GATA-3 IVS4 +1468 SNP showed better CAL reduction and attachment gain than the wild type after non-surgical therapy and 2 years of maintenance. Testing the genotype status in the future might be valuable for individualizing SPT. The results for IL-1, IL-4 and COX-2 polymorphisms were inconsistent as regards the risk for further CAL loss in a well-maintained patient group. Further prospective studies are required to confirm these results.

## Figures and Tables

**Table 1:** Patient characteristics.

	Total Genotyped Patients *n* = 209	Placebo Group *n* = 104	Antibiotics Group *n* = 105	*p*-Value
**Sex; *n* (%)**				
*Male*	116 (55.5%)	58 (55.8%)	58 (55.2%)	1.000 ^F^
*Female*	93 (44.5%)	46 (44.2%)	47 (44.8%)	
**Age; years**	53.1 ±10.0	53.0 ±10.6	53.3 ±9.5	0.929 ^U^
**Active smokers; *n***	45 (21.5%)	19 (18.3%)	26 (24.8%)	0.313 ^F^
**CO ≥ 7 ppm (smoker); *n***	37 (17.7%)	16 (15.7%)	19 (18.1%)	0.712 ^F^
**Diabetes mellitus type II; *n***	12 (5.7%)	6 (5.8%)	6 (5.7%)	1.000 ^F^

Categorical variables are reported as absolute and relative frequencies. Continuous variables are shown as mean ± standard deviation. *p*-values are from Fisher’s exact test ^F^ or Mann–Whitney U test ^U^. Abbreviations: CO: carbon monoxide in exhaled air.

**Table 2:** Genotype frequencies.

Gene Loci	Genotype	Number of Patients; *n* (%)	Placebo Group Patients; *n* (%)	Antibiotics Group Patients; *n* (%)
rs1800587	C/C	102 (48.8%)	49 (47.1%)	53 (50.5%)
IL-1A −889C > T	C/T	87 (41.6%)	46 (44.2%)	41 (39%)
	T/T	20 (9.6%)	9 (8.7%)	11 (10.5%)
	Allele T = MAF	30.9%		
rs1143634	C/C	128 (61.2%)	64 (61.5%)	64 (61%)
IL-1B +3954C > T	C/T	64 (30.6%)	31 (29.8%)	33 (31.4%)
	T/T	17 (8.1%)	9 (8.7%)	8 (7.6%)
	Allele T = MAF	28.5%		
rs2070874	C/C	155 (74.2%)	76 (73.1%)	79 (75.2%)
IL-4 −34C > T	C/T	48 (23%)	26 (25%)	22 (21%)
	T/T	6 (2.9%)	2 (1.9%)	4 (3.8%)
	Allele T = MAF	16.9%		
rs2243250	C/C	154 (73.7%)	76 (73.1%)	78 (74.3%)
IL-4 −590C > T	C/T	48 (23%)	26 (25%)	22 (21%)
	T/T	7 (3.4%)	2 (1.9%)	5 (4.8%)
	Allele T = MAF	18.3%		
rs3802604	G/G	35 (16.8%)	17 (16.3%)	18 (17.1%)
GATA-3	G/A	89 (42.6%)	51 (49%)	38 (36.2%)
IVS4 +1468G > A	A/A	85 (40.7%)	36 (34.6%)	49 (46.7%)
	Allele G = MAF	40.9%		
rs689466	A/A	138 (66.0%)	69 (66.3%)	69 (65.7%)
COX-2 −1195A > G	A/G	64 (30.6%)	32 (30.8%)	32 (30.5%)
	G/G	7 (3.4%)	3 (2.9%)	4 (3.8%)
	Allele G = MAF	18.3%		

Note: Because of rounding of the values, the percentages do not always add up to 100%. Abbreviations: MAF: minor allele frequency.

**Table 3:** Clinical measurements of the change of the CAL.

	Genotype	Total Group Patients *n* CAL Median (25% Quantile, 75% Quantile)	*p*-Value	Placebo Group Patients *n* CAL Median (25% Quantile, 75% Quantile)	*p*-Value	Antibiotics Group Patients *n* CAL Median (25% Quantile, 75% Quantile)	*p*-Value
CAL (mm)	**IL-1A −889 (rs1800587)**						
Change 27.5 months vs. baseline	HG (C/T)	87 −0.5 (−0.8, −0.2)	0.4633	46 −0.4 (−0.8, −0.2)	0.1427	41 −0.6 (−0.9, −0.2)	0.9515
	SNP (T/T)	20 −0.4 (−0.9, 0.1)	0.7852	9 −0.4 (−0.7, 0.2)	0.9319	11 −0.4 (−1.2, 0.0)	0.5832
	WT (C/C)	102 −0.4 (−0.9, 0.0)	ref.	49 −0.2 (−0.7, 0.1)	ref.	53 −0.6 (−1.0, −0.3)	ref.
Change 27.5 months vs. 3.5 months	HG (C/T)	87 0.0 (−0.3, 0.3)	0.1434	46 0.0 (−0.5, 0.2)	**0.0444**	41 0.0 (−0.2, 0.4)	0.9599
	SNP (T/T)	20 0.1 (−0.3, 0.3)	0.9804	9 −0.2 (−0.4, 0.2)	0.4799	11 0.2 (0.0, 0.3)	0.3103
	WT (C/C)	100 0.1 (−0.3, 0.5)	ref.	48 0.2 (−0.1, 0.5)	ref.	52 0.0 (−0.3, 0.4)	ref.
CAL (mm)	**IL−1B +3954 (rs1143634)**						
Change 27.5 months vs. baseline	HG (C/T)	64 −0.4 (−0.8, −0.1)	0.3594	31 −0.3 (−0.6, 0.0)	0.5399	33 −0.6 (−0.9, −0.1)	0.5616
	SNP (T/T)	17 −0.4 (−1.0, −0.2)	0.8660	9 −0.7 (−1.0, −0.4)	0.1375	8 −0.2 (−0.8, 0.1)	0.1920
	WT (C/C)	128 −0.5 (−0.9, 0.0)	ref.	64 −0.4 (−0.8, 0.1)	ref.	64 −0.6 (−1.0, −0.3)	ref.
Change 27.5 months vs. 3.5 months	HG (C/T)	64 0.0 (−0.1, 0.4)	0.3037	31 0.0 (−0.1, 0.5)	0.6826	33 0.0 (−0.1, 0.4)	0.2695
	SNP (T/T)	17 0.1 (−0.3, 0.2)	0.9429	9 −0.3 (−0.4, −0.2)	0.0898	8 0.3 (0.1, 0.6)	**0.0427**
	WT (C/C)	126 0.0 (−0.4, 0.4)	ref.	63 0.1 (−0.5, 0.5)	ref.	63 0.0 (−0.4, 0.4)	ref.
CAL (mm)	**IL−4 −590 (rs2243250)**						
Change 27.5 months vs. baseline	HG (C/T)	48 −0.4 (−0.9, 0.0)	0.7252	26 −0.3 (−0.7, 0.1)	0.6487	22 −0.5 (−1.1, −0.2)	0.9109
	SNP (T/T)	7 −0.6 (−0.8, −0.5)	0.5598	2 −0.7 (−0.8, −0.5)	0.3874	5 −0.6 (−0.7, −0.6)	0.9316
	WT (C/C)	154 −0.4 (−0.9, 0.0)	ref.	76 −0.4 (−0.8, 0.0)	ref.	78 −0.6 (−0.9, −0.1)	ref.
Change 27.5 months vs. 3.5 months	HG (C/T)	47 0.2 (−0.1, 0.4)	0.0864	26 0.2 (−0.1, 0.5)	0.3095	21 0.2 (−0.1, 0.3)	0.1156
	SNP (T/T)	7 0.3 (−0.1, 0.5)	0.3824	2 0.2 (0.1, 0.3)	0.5553	5 0.4 (−0.1, 0.5)	0.5483
	WT (C/C)	153 0.0 (−0.4; 0.4)	ref.	75 0.0 (−0.4, 0.5)	ref.	78 0.0 (−0.3, 0.4)	ref.
CAL (mm)	**GATA−3 IVS4 +1468 (rs3802604)**						
Change 27.5 months vs. baseline	HG (C/T)	89 −0.5 (−0.9, 0.0)	0.1322	51 −0.4 (−0.9, 0.2)	0.1308	38 −0.6 (−0.9, −0.2)	0.6191
	SNP (T/T)	85 −0.5 (−0.9, −0.2)	**0.0184**	36 −0.5 (−0.8, −0.2)	**0.0085**	49 −0.6 (−1.1, −0.1)	0.4352
	WT (C/C)	35 −0.3 (−0.7, 0.0)	ref.	17 −0.1 (−0.3, 0.0)	ref.	18 −0.4 (−0.9, −0.1)	ref.
Change 27.5 months vs. 3.5 months	HG (C/T)	88 0.0 (−0.3, 0.4)	**0.0473**	51 0.0 (−0.4, 0.5)	0.1344	37 0.0 (−0.3, 0.2)	0.3691
	SNP (T/T)	84 0.0 (−0.4, 0.4)	**0.0172**	35 −0.1 (−0.5, 0.4)	**0.0306**	49 0.0 (−0.3, 0.4)	0.2255
	WT (C/C)	35 0.2 (0.0, 0.5)	ref.	17 0.2 (0.1, 0.5)	ref.	18 0.0 (−0.1, 0.5)	ref.
CAL (mm)	**COX−2 −1195 (rs689466)**						
Change 27.5 months vs. baseline	HG (C/T)	64 −0.4 (−0.7, −0.1)	0.1231	32 −0.3 (−0.7, 0.0)	0.4053	32 −0.4 (−0.8, −0.2)	0.1609
	SNP (T/T)	7 −0.5 (−0.7, 0.0)	0.7789	3 −0.5 (−2.0, 0.0)	0.5373	4 −0.4 (−0.7, 0.1)	0.2534
	WT (C/C)	138 −0.5 (−0.9, 0.0)	ref.	69 −0.4 (−0.8, 0.0)	ref.	69 −0.7 (−1.1, −0.1)	ref.
Change 27.5 months vs. 3.5 months	HG (C/T)	64 0.1 (−0.3, 0.5)	0.2653	32 0.0 (−0.3, 0.6)	0.5883	32 0.1 (−0.3, 0.5)	0.2533
	SNP (T/T)	7 0.2 (−0.2, 0.8)	0.4415	3 0.4 (−0.9, 0.8)	0.5986	4 0.1 (−0.1, 0.6)	0.5819
	WT (C/C)	136 0.0 (−0.3, 0.3)	ref.	68 0.1 (−0.5, 0.4)	ref.	68 0.0 (−0.2, 0.3)	ref.

Results are from *n* = 209 patients (placebo *n* = 104, antibiotics *n* = 105) and reported as median (25% quantile, 75% quantile) for continuous variables. *p*-values are from Mann–Whitney U-tests for the pairwise comparison with the WT group. Negative values represent an improvement and positive values represent a deterioration. Abbreviations: CAL: mean clinical attachment level per patient, HG: heterozygote, SNP: single nucleotide polymorphism, WT: wild type, vs.: versus, mm: millimeter, ref.: reference category for pairwise comparison.

**Table 4:** Clinical measurements of the change of the bleeding on probing (BOP).

	Genotype	Total Group Patients *n* BOP Median (25% Quantile, 75% Quantile)	*p*-Value	Placebo Group Patients *n* BOP Median (25% Quantile, 75% Quantile)	*p*-Value	Antibiotics Group Patients *n* BOP Median (25% Quantile, 75% Quantile)	*p*-Value
BOP (%)	**IL-1A −889 (rs1800587)**						
Change 27.5 months vs. baseline	HG (C/T)	87 −21.0 (−33.3, −7.5)	0.5562	46 −13.3 (−29.2, −3.9)	0.5553	41 −24.2 (−42.3, −14.2)	0.7237
	SNP (T/T)	20 −17.1 (−31.6, −3.0)	0.9176	9 −11.9 (−2649.4, −3.0)	0.9489	11 −22.2 (−35.5, −1.2)	0.7972
	WT (C/C)	102 −19.8 (−33.9, −8.3)	ref.	49 −10.8 (−26.8, −7.9)	ref.	53 −23.5 (−39.9, −9.7)	ref.
Change 27.5 months vs. 3.5 months	HG (C/T)	87 0.6 (−7.3, 6.1)	0.0914	46 0.8 (−6.7, 7.2)	0.2574	41 0.6 (−7.3, 5.0)	0.1697
	SNP (T/T)	20 0.7 (−7.2, 5.6)	0.2476	9 0.8 (−7.1, 5.6)	0.2424	11 0.0 (−7.2, 5.6)	0.6326
	WT (C/C)	102 3.0 (−3.5, 10.4)	ref.	49 3.3 (−2.7, 13.3)	ref.	53 2.3 (−3.6, 10.0)	ref.
BOP (%)	**IL-1B +3954 (rs1143634)**						
Change 27.5 months vs. baseline	HG (C/T)	64 −17.7 (−28.6, −3.4)	0.3445	31 −10.9 (−26.4, −2.2)	0.3066	33 −22.7 (−35.5, −14.2)	0.5240
	SNP (T/T)	17 −11.9 (−33.9, −3.0)	0.4081	9 −11.9 (−29.2, −4.9)	0.9267	8 −12.7 (−38.6, 0.6)	0.3191
	WT (C/C)	128 −20.4 (−34.1, −9.3)	ref.	64 −12.2 (−29.4, −8.3)	ref.	64 −24.5 (−42.6, −13.5)	ref.
Change 27.5 months vs. 3.5 months	HG (C/T)	64 1.8 (−6.8, 6.9)	0.6668	31 5.6 (−4.8, 16.0)	0.3805	33 −0.7 (−7.3, 5.0)	0.1349
	SNP (T/T)	17 0.0 (−7.2, 5.6)	0.2950	9 0.7 (−15.4, 5.6)	0.3056	8 −0.6 (−5.7, 10.5)	0.6364
	WT (C/C)	128 1.9 (−5.0, 10.2)	ref.	64 2.2 (−5.2, 10.0)	ref.	64 1.6 (−4.0, 10.2)	ref.
BOP (%)	**IL-4 −590 (rs2243250)**						
Change 27.5 months vs. baseline	HG (C/T)	48 −22.2 (−28.9, −8.6)	1.0	26 −13.3 (−27.0, −6.5)	0.9359	22 −25.3 (−44.9, −14.9)	0.9009
	SNP (T/T)	7 −20.8 (−36.4, 6.9)	0.8848	2 −27.0 (−33.1, −20.8)	0.3380	5 −9.9 (−36.4, 6.9)	0.3366
	WT (C/C)	154 −19.0 (−34.0, −8.2)	ref.	76 −11.5 (−28.6, −3.0)	ref.	78 −22.8 (−39.9, −12.6)	ref.
Change 27.5 months vs. 3.5 months	HG (C/T)	48 2.3 (−2.6, 14.3)	0.2807	26 3.1 (−2.7, 16.0)	0.4220	22 0.6 (−2.6, 13.5)	0.4607
	SNP (T/T)	7 4.2 (−3.7, 14.7)	0.4419	2 8.7 (2.8, 14.7)	0.3704	5 4.2 (−3.7, 5.6)	0.7171
	WT (C/C)	154 1.5 (−6.5, 9.3)	ref.	76 1.8 (−6.9, 9.6)	ref.	78 0.9 (−6.3, 6.7)	ref.
BOP (%)	**GATA-3 IVS4 +1468 (rs3802604)**						
Change 27.5 months vs. baseline	HG (C/T)	89 −20.0 (−33.3, −9.7)	0.1889	51 −14.1 (−33.3, −7.2)	0.0912	38 −21.1 (−34.4, −14.3)	0.8545
	SNP (T/T)	85 −20.2 (−31.3, −8.7)	0.2245	36 −10.7 (−25.7, −4.6)	0.5764	49 −25.0 (−40.2, −14.2)	0.5266
	WT (C/C)	35 −10.0 (−35.5, 3.2)	ref.	17 −9.8 (−24.1, 0.5)	ref.	18 −26.3 (−43.1, 3.2)	ref.
Change 27.5 months vs. 3.5 months	HG (C/T)	89 2.5 (−4.5, 9.7)	0.7126	51 1.8 (−4.5, 9.3)	0.2068	38 4.2 (−5.1, 10.0)	0.4486
	SNP (T/T)	85 0.0 (−5.9, 5.6)	0.3605	36 0.6 (−6.3, 6.6)	0.1617	49 0.0 (−4.1, 5.0)	0.8602
	WT (C/C)	35 4.2 (−7.3, 13.5)	ref.	17 11.1 (−7.3, 18.8)	ref.	18 −0.7 (−6.3, 9.0)	ref.
BOP (%)	**COX-2 −1195 (rs689466)**						
Change 27.5 months vs. baseline	HG (C/T)	64 −18.0 (−33.0, −4.1)	0.5439	32 −10.1 (−27.2, −2.1)	0.4474	32 −26.1 (−40.0, −7.3)	0.9188
	SNP (T/T)	7 −24.5 (−36.3, −14.2)	0.5714	3 −36.3 (−36.8, −24.1)	0.0670	4 −19.4 (−25.9, 1.6)	0.3538
	WT (C/C)	138 −19.4 (−33.9, −9.5)	ref.	69 −12.2 (−26.9, −6.2)	ref.	69 −22.2 (−41.0, −12.8)	ref.
Change 27.5 months vs. 3.5 months	HG (C/T)	64 −0.3 (−7.1, 5.3)	0.1567	32 0.3 (−4.2, 5.7)	0.5034	32 −0.6 (−13.8, 4.9)	0.1588
	SNP (T/T)	7 0.6 (−8.8, 16.7)	0.6159	3 −8.6 (−15.5, 2.5)	0.1472	4 8.7 (−4.1, 17.9)	0.4621
	WT (C/C)	138 2.4 (−4.3, 9.7)	ref.	69 3.3 (−5.4, 11.1)	ref.	69 1.5 (−3.5, 9.0)	ref.

Results are from *n* = 209 patients (placebo *n* = 104, antibiotics *n* = 105) and reported as median (25% quantile, 75% quantile) for continuous variables. *p*-value from Mann–Whitney U-tests. Abbreviations: BOP: percentage of sites showing bleeding on probing per patient, HG: heterozygote, SNP: single nucleotide polymorphism, WT: wild type, vs.: versus.

**Table 5:** Clinical measurements of the change of the percentage of sites with plaque per patient (PS).

	Genotype	Total Group Patients *n* PS Median (25% Quantile, 75% Quantile)	*p*-Value	Placebo Group Patients *n* PS Median (25% Quantile, 75% Quantile)	*p*-Value	Antibiotics Group Patients *n* PS Median (25% Quantile, 75% Quantile)	*p*-Value
PS (%)	**IL-1A −889 (rs1800587)**						
Change 27.5 months vs. baseline	HG (C/T)	87 −2.9 (−19.2, 15.0)	0.8510	46 −3.3 (−18.5, 9.2)	0.4561	41 0.0 (−20.4, 17.6)	0.3430
	SNP (T/T)	20 5.3 (−16.3, 20.1)	0.2619	9 3.6 (−16.9, 17.7)	0.9489	11 12.6 (−15.6, 22.5)	0.1755
	WT (C/C)	102 −1.3 (−20.8, 10.6)	ref.	49 1.8 (−18.6, 12.2)	ref.	53 −10.2 (−21.4, 9.2)	ref.
Change 27.5 months vs. 3.5 months	HG (C/T)	87 9.0 (−2.9, 22.5)	0.4696	46 8.3 (−2.9, 22.5)	0.5073	41 11.5 (−5.4, 21.6)	0.7198
	SNP (T/T)	20 5.5 (−6.8, 27.7)	0.6811	9 −3.4 (−10.7, 6.0)	0.3910	11 23.3 (0.9, 46.3)	0.1357
	WT (C/C)	100 8.4 (−5.7, 19.2)	ref.	48 8.4 (−9.6, 19.2)	ref.	52 8.4 (−2.5, 19.1)	ref.
PS (%)	**IL-1B +3954 (rs1143634)**						
Change 27.5 months vs. baseline	HG (C/T)	64 0.4 (−16.0, 15.4)	0.5137	31 −2.9 (−17.6, 9.2)	0.4768	33 7.1 (−15.6, 17.6)	0.1002
	SNP (T/T)	17 0.0 (−19.2, 17.7)	0.7731	9 −5.0 (−19.2, 17.7)	0.9002	8 2.2 (−22.4, 17.5)	0.5926
	WT (C/C)	128 −3.0 (−20.6, 10.3)	ref.	64 0.7 (−18.5 17.3)	ref.	64 −7.8 (−24.0, 8.2)	ref.
Change 27.5 months vs. 3.5 months	HG (C/T)	64 9.3 (−3.8, 19.9)	0.8409	31 9.0 (−5.9, 21.2)	0.9584	33 9.5 (0.8, 17.9)	0.7996
	SNP (T/T)	17 6.0 (−5.2, 28.3)	0.7627	9 5.0 (−3.4, 10.0)	0.7473	8 25.8 (−8.5, 37.8)	0.3078
	WT (C/C)	126 7.3 (−3.9, 20.0)	ref.	63 7.1 (−5.4, 22.5)	ref.	63 7.9 (−2.6, 20.0)	ref.
PS (%)	**IL-4 −590 (rs2243250)**						
Change 27.5 months vs. baseline	HG (C/T)	48 −4.9 (−20.6, 12.6)	0.3825	26 −4.9 (−19.2, 9.0)	0.1833	22 −7.0 (−21.4, 17.2)	0.9603
	SNP (T/T)	7 −21.4 (−50.0, 3.0)	0.0773	2 −35.7 (−50.0, −21.4)	0.0889	5 −6.9 (−34.3, 3.0)	0.4075
	WT (C/C)	154 0.4 (−18.5, 13.0)	ref.	76 1.7 (−17.2, 22.4)	ref.	78 −1.2 (−19.2, 11.1)	ref.
Change 27.5 months vs. 3.5 months	HG (C/T)	47 7.0 (−6.3, 21.2)	0.7473	26 9.1 (−5.2, 21.2)	0.8102	21 6.5 (−7.1, 19.6)	0.5087
	SNP (T/T)	7 −1.0 (−15.3, 20.0)	0.4539	2 −8.1 (−15.3, −1.0)	0.2335	5 11.1 (−2.8, 20.0)	0.8263
	WT (C/C)	153 8.8 (−3.6, 21.6)	ref.	75 7.4 (−5.4, 19.7)	ref.	78 10.1 (0.0, 22.0)	ref.
PS (%)	**GATA-3 IVS4 +1468 (rs3802604)**						
Change 27.5 months vs. baseline	HG (C/T)	89 0.8 (−21.4, 17.7)	0.9669	51 −3.2 (−23.4, 22.4)	0.2672	38 3.4 (−15.3, 17.6)	0.2150
	SNP (T/T)	85 −1.9 (−19.1, 8.3)	0.4561	36 1.0 (−13.5, 9.4)	0.4665	49 −10.3 (−20.4, 7.1)	0.8768
	WT (C/C)	35 −2.9 (−17.6, 15.5)	ref.	17 0.0 (−7.8, 22.6)	ref.	18 −5.7 (−30.2, 15.2)	ref.
Change 27.5 months vs. 3.5 months	HG (C/T)	88 9.7 (−3.8, 20.4)	0.5387	51 6.0 (−5.9, 19.7)	0.6878	37 13.6 (5.6, 21.6)	0.1354
	SNP (T/T)	84 6.1 (−5.4, 22.5)	0.7468	35 9.4 (−1.5, 18.1)	0.9690	49 4.5 (−5.6, 23.3)	0.6778
	WT (C/C)	35 6.5 (−6.9, 18.5)	ref.	17 4.6 (−2.9, 24.0)	ref.	18 6.8 (−8.7, 15.1)	ref.
PS (%)	**COX-2 −1195 (rs689466)**						
Change 27.5 months vs. baseline	HG (C/T)	64 −6.5 (−21.1, 12.8)	0.2872	32 −8.3 (−25.0, 13.7)	0.0985	32 −1.2 (−17.1, 12.8)	0.9333
	SNP (T/T)	7 −17.6 (−20.4, 9.2)	0.5683	3 4.5 (−17.6, 9.2)	1.0	4 −19.7 (−37.1, 22.2)	0.5072
	WT (C/C)	138 0.6 (−19.2, 14.3)	ref.	69 1.5 (−17.6, 15.0)	ref.	69 −0.5 (−22.0, 13.0)	ref.
Change 27.5 months vs. 3.5 months	HG (C/T)	64 7.7 (−6.0, 22.3)	0.8578	32 10.2 (−4.4, 22.7)	0.9061	32 6.8 (−7.7, 20.5)	0.6826
	SNP (T/T)	7 8.8 (−5.6, 46.3)	0.6440	3 4.6 (−20.4, 18.8)	0.7650	4 27.5 (1.6, 49.4)	0.3471
	WT (C/C)	136 8.0 (−3.3, 19.8)	ref.	68 4.4 (−5.3, 18.3)	ref.	68 10.2 (−0.2, 21.0)	ref.

Results are from *n* = 209 patients (placebo *n* = 104, antibiotics *n* = 105) and reported as median (25% quantile, 75% quantile) for continuous variables. *p*-value from Mann–Whitney U-tests. Abbreviations: PS: percentage of sites with plaque per patient, HG: heterozygote, SNP: single nucleotide polymorphism, WT: wild type, vs.: versus.

**Table 6:** Multivariable linear model estimates for the change of mean CAL from 27.5 months versus baseline and from 27.5 months versus 3.5 month.

		Dependent Variable							
		Change of Mean CAL (27.5 Month Baseline) Per Patient				Change of Mean (27.5 Month - 3.5 Month) Per Patient			
Independent Variables		β	95% CI		*p*-Value	β	95% CI		*p*-Value
*Intercept*		−0.39	−0.64	−0.13	0.0027	0.22	0.01	0.43	0.0393
**Therapy**	Placebo vs. AB	0.22	0.04	0.39	**0.0132**	−0.03	−0.18	0.11	0.6509
**IL-1A** **-889**	Global				0.1800				0.0878
	HG vs. WT	−0.17	−0.39	0.04	0.1281	−0.17	−0.36	0.0	0.0574
	SNP vs. WT	0.06	−0.36	0.49	0.7801	0.03	−0.32	0.38	0.8654
**IL-1B** **+3954**	Global				0.1117				0.3163
	HG vs. WT	0.20	−0.03	0.44	0.1010	0.13	−0.06	0.33	0.1833
	SNP vs. WT	−0.12	−0.57	0.31	0.5754	−0.02	−0.39	0.33	0.8816
**IL-4 −34**	Global				0.4058				0.2384
	HG vs. WT	0.86	−0.43	2.15	0.1903	0.17	−0.89	1.24	0.7425
	SNP vs. WT	0.65	−1.21	2.52	0.4915	1.12	−0.41	2.67	0.1518
**IL-4 −590**	Global				0.4114				0.3364
	HG vs. WT	−0.86	−2.14	0.42	0.1886	−0.07	−1.13	0.99	0.8961
	SNP vs. WT	−0.72	−2.54	1.08	0.4298	−0.86	−2.36	0.64	0.2600
**GATA-3** **+1468**	Global				**0.0355**				0.0605
	HG vs. WT	−0.18	−0.43	0.07	0.1591	−0.21	−0.42	0.0	**0.0424**
	SNP vs. WT	−0.32	−0.57	−0.07	**0.0116**	−0.24	−0.45	−0.03	**0.0218**
**COX-2** **-1195**	Global				0.3306				0.4510
	HG vs. WT	0.14	−0.04	0.33	0.1384	0.09	−0.06	0.25	0.2523
	SNP vs. WT	0.02	−0.47	0.51	0.9261	0.14	−0.26	0.55	0.4935

Results are from *n* = 209 patients. Intercept: Therapy = Antibiotics. IL-1A −889 = WT, IL-1B +3954 = WT, IL-4 −34 = WT, IL-4 −590 = WT, GATA-3 +1468 = WT, and COX-2-1195 = WT. For example a placebo patient with IL-1A −889 = HG, IL-1B +3954 = SNP, IL-4 −34 = HG, IL-4 −590 = SNP, GATA-3 +1468= SNP, and COX-2-1195= WT has an expected change of the mean CAL between 27.5 months and baseline of: −0.39 + 0.22 − 0.17 − 0.12 + 0.86 − 0.72 − 0.32 + 0 = −0.64 mm. *p*-values for pairwise comparisons are from the Wald Tests and global *p*-values are from the F-Test. Abbreviations: AB = Antibiotic, HG = Heterozygous genotype, SNP = Single nucleotide polymorphism, WT: wild type, β = regression coefficient (least square mean estimate), CI = Confidence interval (lower limit, upper limit).

## Data Availability

The raw data and other related data in the manuscript are available from the corresponding author, K.-A.W., upon reasonable request.

## Supplementary Materials

The following supporting information can be downloaded at: https://www.mdpi.com/article/10.3390/ijms23137266/s1.
