# Supplementary material for: The Role of Polymorphisms at the Interleukin-1, Interleukin-4, GATA-3 and Cyclooxygenase-2 Genes in Non-Surgical Periodontal Therapy
Source: Int J Mol Sci. 2022 Jun 30;23(13):7266. doi: 10.3390/ijms23137266 (PMC9266438; doi:10.3390/ijms23137266)
Supplement: Supplementary file 1 [file ijms-23-07266-s001.zip › ijms-1747666-supplementary.pdf]

Supplementary file

Table S1: Clinical measurements of the change of the percentage of sites showing further attachment loss (PSAL) ≥1.3mm

	Genotype	Total group patients n PSAL ≥1.3mm Median (25% Quantile, 75% Quantile)	*p*-value	Placebo group patients n PSAL ≥1.3mm Median (25% Quantile, 75% Quantile)	*p*-value	Antibiotics group patients n PSAL ≥1.3mm Median (25% Quantile, 75% Quantile)	*p*-value
PSAL ≥1.3mm (%)	**IL-1A -889 (rs1800587)**						
Change 27.5 months vs. baseline	HG (C/T)	87<br>6.7 (3.6, 11.8)	0.2019	46<br>8.4 (4.3, 15.3)	0.5355	41<br>5.8 (3.6, 9.6)	0.2851
	SNP (T/T)	20<br>4.4 (2.2, 11.2)	0. 4905	9<br>5.6 (3.2, 10.1)	0.6231	11<br>3.6 (2.1, 12.3)	0.5710
	WT (C/C)	102<br>5.6 (3.1, 9.5)	ref.	49<br>6.1 (3.7, 13.5)	ref.	53<br>4.5 (2.7, 7.5)	ref.
Change 27.5 months vs. 3.5 months	HG (C/T)	87<br>9.1 (4.8, 14.6)	0.1432	46<br>11.0 (5.2, 16.7)	0.2167	41<br>8.0 (4.7, 11.9)	0.4848
	SNP (T/T)	20<br>8.8 (3.5, 11.8)	0.9285	9<br>7.9 (4.7, 11.9)	0.8307	11<br>9.7 (3.0, 11.7)	0.9013
	WT (C/C)	100<br>7.8 (4.4, 12.8)	ref.	48<br>8.7 (4.9, 13.2)	ref.	52<br>7.3 (3.7, 11.5)	ref.
PSAL ≥1.3mm (%)	**IL-1B +3954 (rs1143634)**						
Change 27.5 months vs. baseline	HG (C/T)	64<br>5.8 (3.3, 12.2)	0.8440	31<br>7.1 (3.7, 17.3)	0.7848	33<br>4.8 (3.2, 9.5)	0.8793
	SNP (T/T)	17<br>5.6 (3.2, 15.0)	0.7825	9<br>5.6 (3.2, 11.7)	0.3591	8<br>4.9 (3.0, 15.5)	0.6749
	WT (C/C)	128<br>5.6 (3.6, 10.3)	ref.	64<br>6.5 (4.1, 14.2)	ref.	64<br>5.0 (2.6, 8.3)	ref.
Change 27.5 months vs. 3.5 months	HG (C/T)	64<br>9.0 (4.7, 14.0)	0.2914	31<br>10.8 (4.7, 20.3)	0.4792	33<br>8.0 (4.9, 11.7)	0.3322
	SNP (T/T)	17<br>10.3 (3.6, 19.4)	0.4221	9<br>10.3 (3.9, 12.0)	0.8804	8<br>10.8 (3.3, 20.0)	0.3733
	WT (C/C)	126<br>8.0 (4.6, 12.6)	ref.	63<br>9.7 (5.4, 13.5)	ref.	63<br>7.3 (3.9, 11.2)	ref.
PSAL ≥1.3mm (%)	**IL-4 -590 (rs2243250)**						
Change	HG (C/T)	48	**0.0225**	26	0.1240	22	0.1320

27.5 months vs. baseline		8.3 (4.4, 15.7)		11.2 (4.4, 17.3)		6.8 (4.0, 10.9)	
	SNP (T/T)	7<br>3.1 (1.4, 6.5)	0.1720	2<br>8.9 (6.5, 11.3)	0.5498	5<br>2.1 (1.4, 3.1)	**0.0488**
	WT (C/C)	154<br>5.4 (3.3, 10.0)	ref.	76<br>5.8 (3.6, 13.8)	ref.	78<br>4.7 (2.8, 8.3)	ref.
Change 27.5 months vs. 3.5 months	HG (C/T)	47<br>11.4 (5.9, 17.7)	**0.0202**	26<br>11.8 (7.1, 17.5)	0.0907	21<br>10.1 (4.2, 17.9)	0.1462
	SNP (T/T)	7<br>6.2 (5.6, 11.1)	0.8393	2<br>11.6 (11.1, 12.0)	0.4318	5<br>6.1 (5.6, 6.2)	0.4750
	WT (C/C)	153<br>8.0 (3.8; 12.0)	ref.	75<br>9.1 (4.0, 13.4)	ref.	78<br>7.7 (3.6, 11.1)	ref.
PSAL ≥1.3mm (%)	**GATA-3 IVS4 +1468 (rs3802604)**						
Change 27.5 months vs. baseline	HG (C/T)	89<br>5.6 (3.3, 12.3)	0.0961	51<br>5.8 (3.6, 13.5)	0.0743	38<br>5.1 (3.3, 9.9)	0.5245
	SNP (T/T)	85<br>5.2 (2.9, 9.4)	**0.0156**	36<br>6.0 (3.3, 11.6)	0.0855	49<br>4.2 (2.4, 6.7)	0.1557
	WT (C/C)	35<br>9.0 (4.3, 15.1)	ref.	17<br>10.0 (5.8, 16.1)	ref.	18<br>5.7 (2.6, 12.7)	ref.
Change 27.5 months vs. 3.5 months	HG (C/T)	88<br>8.0 (4.7, 12.0)	0.1162	51<br>9.5 (4.8, 13.2)	0.1126	37<br>7.7 (4.2, 11.7)	0.4082
	SNP (T/T)	84<br>7.9 (4.2, 12.3)	0.0551	35<br>9.1 (4.0, 13.5)	0.1242	49<br>7.7 (4.4, 10.8)	0.3649
	WT (C/C)	35<br>10.7 (5.3, 18.5)	ref.	17<br>11.8 (9.1, 18.5)	ref.	18<br>9.8 (3.2, 18.3)	ref.
PSAL ≥1.3mm (%)	**COX-2 -1195 (rs689466)**						
Change 27.5 months vs. baseline	HG (C/T)	64<br>5.3 (3.2, 12.3)	0.5920	32<br>6.1 (3.0, 13.1)	0.3179	32<br>4.7 (3.3, 10.0)	0.6809
	SNP (T/T)	7<br>6.8 (3.7, 10.9)	0.6652	3<br>3.7 (3.6, 6.8)	0.2139	4<br>9.6 (6.6, 15.9)	0.0769
	WT (C/C)	138<br>5.8 (3.3, 10.8)	ref.	69<br>7.4 (4.4, 15.1)	ref.	69<br>4.8 (2.6, 8.3)	ref.
Change 27.5 months vs. 3.5 months	HG (C/T)	64<br>8.7 (4.9, 13.8)	0.5456	32<br>8.9 (4.3, 15.3)	0.5412	32<br>8.7 (5.7, 13.4)	0.1267
	SNP (T/T)	7<br>9.5 (6.2, 18.5)	0.5777	3<br>9.5 (6.2, 18.5)	0.8552	4<br>10.0 (4.7, 17.3)	0.5625
	WT (C/C)	136<br>8.2 (4.2, 12.3)	ref.	68<br>10.4 (4.8, 13.9)	ref.	68<br>7.1 (3.3, 10.4)	ref.

Results are from n = 209 patients (placebo n=104, antibiotics n=105) and reported as median (25% quantile, 75% quantile) for continuous variables.

*p-values are from Mann–Whitney U-tests for the pairwise comparison with the WT group.

Negative values represent an improvement and positive values represent a deterioration.

Abbreviations: PSAL ≥1.3mm: percentage of sites showing further attachment loss ≥1.3mm per patient, HG: heterozygote, SNP: single nucleotide polymorphism, WT: wild type, vs.: versus, mm: millimeter, %: percent, ref.: reference category for pairwise comparison.
